# Optimization of Cell Adhesion on Mg Based Implant Materials by Pre-Incubation under Cell Culture Conditions

**DOI:** 10.3390/ijms15057639

**Published:** 2014-05-05

**Authors:** Regine Willumeit, Anneke Möhring, Frank Feyerabend

**Affiliations:** Helmholtz-Zentrum Geesthacht, Institute of Materials Research, Max-Planck-Str. 1, Geesthacht 21502, Germany; E-Mails: anmo@tf.uni-kiel.de (A.M.); frank.feyerabend@hzg.de (F.F.)

**Keywords:** magnesium implants, natural corrosion protection, pre-incubation, biocompatibility

## Abstract

Magnesium based implants could revolutionize applications where orthopedic implants such as nails, screws or bone plates are used because they are load bearing and degrade over time. This prevents a second surgery to remove conventional implants. To improve the biocompatibility we studied here if and for how long a pre-incubation of the material under cell culture conditions is favorable for cell attachment and proliferation. For two materials, Mg and Mg10Gd1Nd, we could show that 6 h pre-incubation are already enough to form a natural protective layer suitable for cell culture.

## 1. Introduction

To make use of magnesium alloys as orthopedic biomaterial is an idea which has developed already in the first half of the 20th century [1]. Mg is an essential element in life and about half of the 30 g which can be found in the body is stored in bones [2]. The material is load bearing and the mechanical properties tensile yield strength, fracture toughness or Young’s modulus [3] are more similar to those of natural bone than polymers, ceramics implant materials or titanium (Ti) which remains the most common and preferred implant material. Last but not least the metal is degrading which should have several advantages over permanent implant materials [4,5]. However, due to their high degradation rates accompanied by hydrogen (H_2_) release up to now only few implant prototypes are about to come for orthopedic [6] or cardiovascular [7,8] applications. The major problem remains the decrease of the degradation rate to keep the stability of the implant and to reduce the ion release and H_2_ production to a value the body can deal with [9].

There are several approaches to tailor the degradation rate. The first step is the use of alloying elements (e.g., rare earth elements (REE [10,11], Ca, Y, Ag *etc.* [12])) accompanied by heat treatments [13] or for example extrusion processes which change the microstructure and thus the mechanical and corrosive properties. If this is still not enough coatings come into play. The principle idea is to reduce the immediate corrosion to a level the body can cope with directly after implantation. Over time also the coating should degrade offering the possibility for the underlying Mg material to have access to the surrounding liquid which will then start the degradation process of the metal.

In the literature several approaches can be found (for an overview see [9,14–16]). The most often used coating is based on calcium phosphates (for review see [17]). This is relative straight forward because a material which is also found in the anorganic matrix of the bone is used as coating. In addition under certain circumstances it is already a corrosion product of the degrading Mg material [18–20]. The application of CaP coatings not only changes the surface chemistry and can be influenced by the presence of chloride salts and proteins [21]. In addition, the micro- [22,23] or nanostructure of the HA crystallites deposited on the surface can improve cell adhesion and reduce the corrosion rate [24]. To improve the performance of the CaP coatings also composites with degradable polymers were prepared [25,26].

Degradable (bio) polymers as such are also a useful approach to increase the resistance of the Mg material against corrosion. They do not only hinder the immediate corrosion but can also influence the long term behavior of the specimens [27]. In addition these layers can be chemically modified to introduce specific properties or can be used as drug delivery systems [28].

A promising alternative to relatively thick polymer coatings can be the covalent binding of proteins [29,30] or for example 3-amino-propyltrimethoxysilane [31] and the lipid head group phosphatidylcholine (PC) [32] to activated Mg-alloy surfaces.

Protection layers can also be formed by a fluoride coating [33,34] or micro-arc oxidation (MAO) [35,36]. Doping the surface with Nd by diffusion coating [37] or treating it with NaOH [38,39] also reduces the corrosion rate.

The most straight forward approach however is to make use of the naturally formed protective layer which is found after some immersion time (for example [40]) and also *in vivo* [41,42]. The formation of this biomimetic coating was studied in several experiments. Mg specimens were either soaked at 37 °C in SBF for 5 days [43], in growth medium DMEM (without and with FBS) or pure FBS for 24 h under cell culture conditions [44]. The results showed that in all cases the cell attachment and proliferation rate was improved with respect to the untreated and thus faster degrading specimens. The best results were obtained for the incubation in DMEM.

In this study, we systematically investigated which pre-incubation time gives the optimal results with respect to cell adhesion, proliferation, corrosion rate and composition of the corrosion layer. We used extruded material and elucidated pure Mg and the alloy Mg10Gd1Nd which have different corrosion rates. All pre-incubation tests were performed in DMEM and under cell culture conditions varying the exposure time between 30 min and three days.

## 2. Results and Discussion

The aim of this study was to elucidate if a time dependent improvement of cell interaction depending on varying pre-incubation times can be observed. Therefore specimens were pre-incubated to form a natural corrosion and probably protective layer. Then the samples were washed, dried and stored until the cell experiments were started. All specimens were incubated with Saos-2 cells for 30 min to achieve cell adhesion. Subsequently all specimens were then incubated for three further days to be examined for the parameters corrosion rate, pH, osmolality, cell viability and shape as well as element distribution on the surface. Thus the total immersion time varied from 72.5 h for untreated specimen (non pre-incubation, only cell adhesion time + cell culture incubation) to 144.5 h after 3 days of pre-incubation.

### 2.1. Determination of the Corrosion Rate, pH and Ssmolality

**Figure 1.**
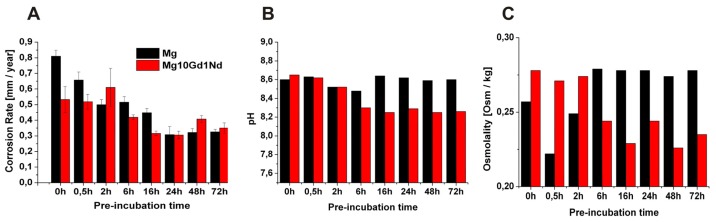
(**A**) Corrosion rate; (**B**) pH values and (**C**) osmolality determined after the indicated pre-incubation time.

The corrosion rate of the two materials without pre-incubation and cells was determined to 0.81 ± 0.04 mm/year for Mg. For the alloy Mg10Gd1Nd this value is two third = 0.53 ± 0.08 mm/year. This was decreased upon pre-incubation to values of 0.33 ± 0.02 and 0.35 ± 0.03 mm/year for Mg and Mg10Gd1Nd (Figure 1A).

To determine the pH values the samples were first pre-incubated in cell culture medium for the indicated duration. Then the specimens were washed, dried and then incubated with cells for 3 days and the pH value at the end of this period was determined (Figure 1B). As it can be seen the pH is increased above the physiological level to 8.6 and nearly the same for both materials when the specimens were not pre-incubated. However, for pre-incubated specimens clear differences can be seen for the materials. In the case of pure extruded Mg the pre-incubation has nearly no influence on the pH observed. It is in all cases around 8.6 which is significantly higher than the pH-value of fresh, pure Mc Coy’s medium which is between 6.9 and 7.1. For the Mg10Gd1Nd alloy a clear decrease in pH depending on the pre-incubation time can be seen. This indicates the formation of a protective layer which does not completely hinder the corrosion process.

The osmolality was measured in the supernatant which was also used for pH determination. The results of this measurement are shown in Figure 1C. The measured osmolality of the supernatants is equal or lower than the cell culture medium which equals to 0.283 (±0.001, *n* = 6). Two trends can be observed: the osmolality of the medium from the extruded Mg10Gd1Nd decreases with longer pre-incubation time while the osmolality of the medium from pure magnesium increases with longer pre-incubation times. During degradation of the material in the absence of cells normally an increase of osmolality can be observed [45]. A drop in osmolality could indicates that the ion release from the degrading material is lower than the consumption of ions by metabolically active cells and could be used as an indirect measure of cell viability.

### 2.2. Determination of Cell Viability

**Figure 2.**
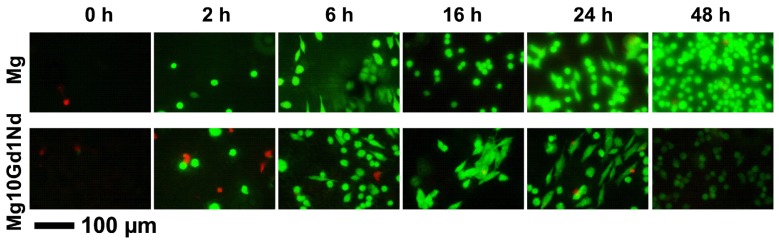
Life Dead Staining of Saos-2 cells incubated on two materials with varying pre-incubation times.

The cells were cultivated for three days on the pre-incubated specimens. It can clearly be seen that the cell viability is zero for specimens which did not experience a pre-incubation (Figure 2). However, after only 2 h one can see the first positive effect. In general a pre-incubation of 6 h is enough to form a surface which is favorable for cell adhesion and proliferation. Longer pre-incubation times optimize this behavior but not in a way that would justify an increased experiment period, as the contamination risk is increasing with time.

### 2.3. Imaging with Scanning Electron Microscope (SEM) and Energy Dispersive X-ray Spectroscopy (EDX) Analysis

**Figure 3.**
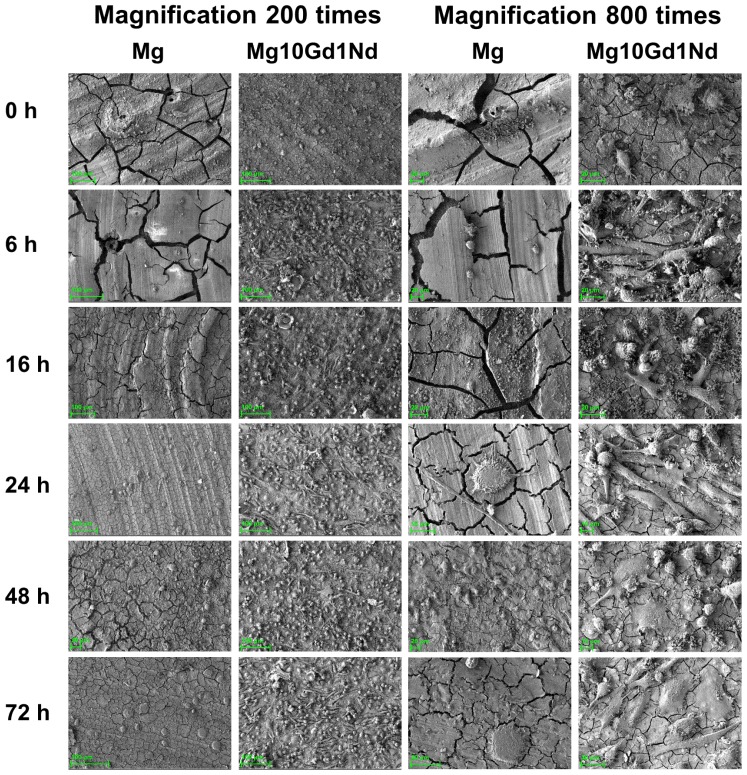
SEM images of Saos-2 cells incubated on two materials with varying pre-incubation times.

While in Figure 2 the viability of cells was imaged, with SEM the cell morphology can be seen in detail (Figure 3). Comparable to the findings in Figure 2 well attached cells are found already after 6 h of pre-incubation time.

**Figure 4.**
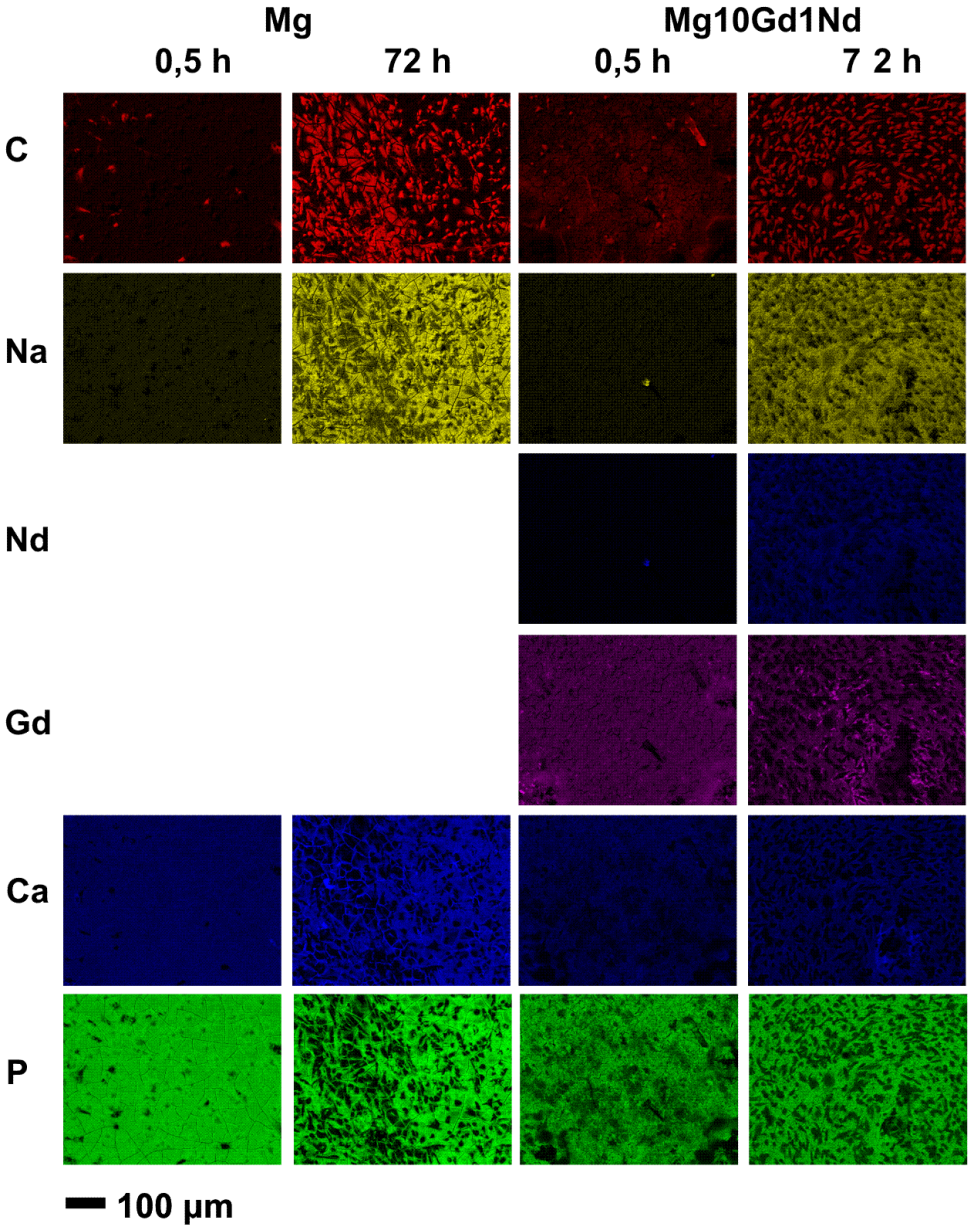
EDX element mapping of specimens with Saos-2 cells incubated on two materials with varying pre-incubation times.

The analysis of the corrosion layer surface by EDX (Figure 4) exhibits the increase of cell attachment (carbon-mapping; C) after 72 h of pre-incubation time. In the case of the alloy the pre-incubation leads to an exposure of the alloying elements. In addition the deposition of Ca and Na coming from the incubation medium is visualized for a longer pre-incubation time. Interestingly P is found already after the short pre-incubation time of 0.5 h. The co-precipitation of Ca and P indicates the formation of calcium-phosphates. A further interesting observation is that there is a similar distribution of Na and Nd, which could indicate that during the corrosion process new complex phases could be formed. This could be e.g., the formation of Na_7_Mg_13_Nd(PO_4_)_12_, which was recently crystallized from a solution containing Na_2_CO_3_, MgCO_3_, Nd_2_O_3_, NH_4_H_2_PO_4_, [46] which are all ingredients of cell culture medium (except for Nd, which is derived from the alloy).

### 2.4. Discussion of Results

In this study, we evaluated the optimal pre-incubation time in cell culture medium for pure Mg and the alloy Mg10Gd1Nd. Both materials exhibit a corrosion rate below 1 mm/year which is in our opinion the maximum corrosion rate suitable for proper *in vivo* application. However, even this low corrosion rate might pose problems in *in vitro* experiments because already then gas formation and increase of pH might prevent cell adhesion and proliferation. Therefore pre-incubation was applied to create a natural corrosion protection layer. Our results show that already 6 h under cell culture conditions are sufficient to form a protective layer suitable for cell experiments. Though the corrosion rate is reduced by this layer formation it does not necessarily change the pH and osmolality values. This indicates that not the mere decrease in corrosion rate facilitates cell adhesion but that probably the combination of reduced gas formation plus a change in surface chemistry and morphology supports cell growth on the material. The results—in terms of cell adhesion and proliferation—are comparable for both materials. This indicates that influence of alloying elements on the cells can be neglected at least in the first few days of experiments.

A detailed study of the composition of the corrosion layer could shed light on the mechanisms of corrosion in this very complex environment and might bridge the gap toward *in vivo* corrosion studies.

## 3. Experimental Section

### 3.1. Materials

Pure magnesium and Mg10Gd1Nd were cast in the Magnesium Innovation Center (MagIC) of the Helmholtz-Zentrum Geesthacht by direct-chill casting. High-purity magnesium (99.94%) was obtained from Magnesium Elektron (Manchester, UK). Pure Gd and Nd (99.5%) were obtained from Grirem (Beijing, China). The nominal amounts were added to the melt (750 °C) under a protective atmosphere (Ar + 2% SF_6_, Linde, Hamburg, Germany), which was stirred at 200 rpm and then poured in a preheated mold (550 °C). The cleanliness of the ingots was assured by using a filter (Foseco SIVEX FC, Foseco, London, UK). Cylindrical ingots with a diameter of 12 cm and a length of 20 cm were obtained. The materials were afterwards extruded in the Strangpresszentrum Berlin to rods with 12 mm diameter and machined to round discs with dimensions of 10 × 1.5 mm. The average weight of the specimens was approximately 0.2 g. They were used without further surface treatment.

Samples were disinfected in 70% ethanol (VWR International, Darmstadt, Germany) in an ultrasonic bath for 20 min. Afterwards they were processed in parallel using standard 24 well plates (Cellstar, Greiner Bio-one GmbH, Frickenhausen, Germany), with one sample per well. Pure Mg and Mg10Gd1Nd discs were directly used (0 h) or immersed in 2 mL Mc Coy’s cell culture medium (Mc Coy’s 5A Medium, Life Technologies, Darmstadt, Germany) supplemented with 10% FBS (FBS Gold, PAA Laboratories, Linz, Austria) for 0.5, 2, 6, 16, 24, 48 h and under cell culture conditions (37 °C, 21% O_2_, 5% CO_2_, 95% relative humidity). In each case *n* = 4 was chosen per alloy and per pre-incubation time.

### 3.2. Determination of Corrosion Rate, pH and Osmolality

The corrosion rate was determined by weight loss. Each sample was weighed (Scaltec SBA52, Scaltec Instruments GmbH, Göttingen, Germany), numbered and sterilized (again 20 min in ultrasonic bath with ethanol) before pre-incubation (*n* = 3 per alloy and pre-incubation time). After pre-incubation the samples were placed into a new agarose coated 12-well-plate and treated as described below with cells. After three days of incubation under cell culture conditions the samples were taken from the corrosion medium. To remove all corrosion residues from the samples they were immersed in chromic acid (180 g/L in distilled water, VWR International, Darmstadt, Germany) for 10 min, turned and left for another 10 min. Afterwards the samples were cleaned with distilled water and then with 100% ethanol. When the samples were dried their weight was measured again and the corrosion rate (CR) was calculated as follows [47]:

$$CR=\frac{8.76\times 10^{4}\times \Delta W}{A\times t\times \rho }$$

*A* = surface area [cm^2^], *t* = time [h], *ρ* = density (Mg = 1.74; Mg10Gd1Nd = 1.90 [g/cm^3^]), Δ*W* = mass loss [g].

After the final three days of incubation with cells on the pre-incubated specimens the cell culture medium was removed from the wells and used for analysis of the pH-value and osmolality. For measuring the pH-value the electrode was calibrated each time before using. Then the electrode was dipped into the solution and left there until the value on the display of the measurement device was stable.

The osmolality of the supernatants was analyzed by cryoscopic osmometer (Osmomat 030-D, Gonotec GmbH, Berlin, Germany). 60 μL of the supernatant were pipetted into a small cup (1 mL) and put into the osmometer. The device is a freezing point osmometer which determines the osmolality by measuring the freezing point. This goes down by 1.86 °C when 1 mole of a non ionic solute is added to one kilogram of solvent [48].

### 3.3. Determination of Cell Viability

The human osteosarcoma cell line Saos-2 was purchased from the European Collection of Cell Cultures (ECACC, Salisbury, UK) and cultured under cell culture conditions.

After pre-incubation, the Mg samples were transferred into agarose pre-coated 12-well-plate (one sample per well). The agarose-coating was applied by heating pre-sterilized agarose (1% *w*/*v* in a dest; VWR International, Darmstadt, Germany) to about 80 °C and rapid filling and decanting of the wells. This leads to a thin film of agarose throughout the well. For cell adhesion, Saos-2 cells were trypsinized, centrifuged and afterwards resuspended in Mc Coy’s medium + 10% FBS to a concentration of 1 × 10^6^ cells per mL. Then 50 μL of the cell suspension were homogeneously applied onto each sample. Samples were incubated for 30 min to allow the study of initial and fast adhesion. Then 2 mL Mc Coy’s medium + 10% FBS was added in each well of the 12-well-plate followed by a further incubation for three days under cell culture conditions.

Cell viability was assessed via a two-color fluorescence assay (Live/Dead Viability/Cytotoxicity Kit, Life Technologies, Darmstadt, Germany). One sample per pre-incubation time was used for live/dead staining. After removing the cell culture medium the sample was washed with phosphate buffer solution (PBS, 137 mM NaCl, 2.7 mM KCl, 4.3 mM Na_2_HPO_4_, 1.4 mM KH_2_PO_4_, all chemicals from VWR), then transferred to a new 12-well-plate and covered with the staining solution (2 μL Calcein AM and 5 μL ethidiumhomidimer-1 in 5 mL PBS) and incubated for 20 min. Afterwards the staining solution was replaced by PBS and samples were directly examined with a fluorescent microscope (Eclipse Ti-S, Nikon, Düsseldorf, Germany). Living cells can be detected by using the FITC-filter (Ex: 465–495 nm, Em: 515–555 nm, Mirror at 505 nm) as green areas, dead cells can be observed by using the Texas Red-filter (Ex: 540–580 nm, Em: 600–660 nm, Mirror at 595 nm) as red areas. The overlaying image was merged by the microscope software.

### 3.4. Imaging with Low-Voltage Scanning Electron Microscope (LVSEM) and Energy Dispersive X-ray Spectroscopy (EDX) Analysis

To visualize magnesium samples covered with cells without the application of additional coatings a special sample preparation was needed. After removing the cell culture medium the samples were washed with PBS and put on a new 12-well-plate (one sample per well). All chemicals were supplied by VWR International. 2.5% glutaraldehyde solution was loaded in each well and the plate was stored overnight under the fume hood. On the next day samples were placed in a new plate and the glutaraldehyde was replaced by 1% osmiumtetroxide for 30 min for lipid counterstaining [49]. Samples were again put into a new well to start the alcoholic row. At first 20% isopropanol was added in each well so that the samples were completely covered. After one to two hours it was replaced by 40% isopropanol for another one to two hours. The same procedure was repeated with 60%, 80%, and finally with 100% isopropanol followed by critical point drying (EM CPD030, Leica Mikrosysteme Vertrieb GmbH, Wetzlar, Germany). During the procedure of critical point drying the isopropanol contained in the cells was slowly and totally replaced by CO_2_ at 8 °C. Afterwards, temperature and pressure were raised to the critical point of CO_2_, leading to water-free preparations. After drying samples were transferred into a new 12-well-plate and stored in a drying cabinet. Cells on carriers were then visualised by low voltage mode in charge contrast at 1 keV, using the SEM in lens detector (SEM Auriga, Carl Zeiss, Jena, Germany).

Additionally to the SEM an analysis with energy dispersive X-ray spectroscopy (EDX) was performed (EDS; Apollo XP, EDAX, Ametek GmbH, Wiesbaden, Germany). The preparation of the samples was done in the same way as for SEM. EDX-mapping of various elements (C, Na, Mg, Nd, Gd, Ca, P) was done at an accelerating voltage of 8 keV with 120 μm aperture size at 200-fold magnification.

## 4. Conclusions

The natural protection layer which is formed already after 6 h of pre-incubation under cell culture conditions is enough to improve cell adhesion and viability. This is—at least for Mg and Mg10Gd1Nd studied here—independent of the material and its corrosion rate.

## References

[1] Witte F. (2010). The history of biodegradable magnesium implants: A review. Acta Biomater.

[2] Vormann J. (2003). Magnesium: Nutrition and metabolism. Mol. Asp. Med.

[3] Staiger M.P., Pietak A.M., Huadmai J., Dias G. (2006). Magnesium and its alloys as orthopedic biomaterials: A review. Biomaterials.

[4] Atrens A., Liu M., Zainal Abidin N.I. (2011). Corrosion mechanism applicable to biodegradable magnesium implants. Mater. Sci. Eng. B.

[5] Song G.L., Atrens A. (1999). Corrosion mechanisms of magnesium alloys. Adv. Eng. Mater.

[6] Windhagen H., Radtke K., Weizbauer A., Diekmann J., Noll Y., Kreimeyer U., Schavan R., Stukenborg-Colsman C., Waizy H. (2013). Biodegradable magnesium-based screw clinically equivalent to titanium screw in hallux valgus surgery: Short term results of the first prospective, randomized, controlled clinical pilot study. BioMed. Eng. OnLine.

[7] Haude M., Erbel R., Erne P., Verheye S., Degen H., Böse D., Vermeersch P., Wijnbergen I., Weissman N., Prati F. (2013). Safety and performance of the drug-eluting absorbable metal scaffold (DREAMS) in patients with *de-novo* coronary lesions: 12 month results of the prospective, multicenter, first-in-man BIOSOLVE-I trial. Lancet.

[8] Erbel R., Böse D., Haude M., Kordish I., Churzidze S., Malyar N., Konorza T., Sack S. (2007). Absorbierbare Stents. Herz.

[9] Virtanen S. (2011). Biodegradable Mg and Mg alloys: Corrosion and biocompatibility. Mater. Sci. Eng. B.

[10] Feyerabend F., Fischer J., Holtz J., Witte F., Willumeit R., Drücker H., Vogt C., Hort N. (2010). Evaluation of short-term effects of rare earth and other elements used in magnesium alloys on primary cells and cell lines. Acta Biomater.

[11] Witte F., Hort N., Vogt C., Cohen S., Kainer K.U., Willumeit R., Feyerabend F. (2008). Degradable biomaterials based on magnesium corrosion. Curr. Opin. Solid State Mater. Sci.

[12] Kainer K.U., Bala Srinivasan P., Blawert C., Dietzel W., Cottis B., Graham M., Lindsay R., Lyon S., Richardson T., Scantlebury D., Stott H. (2010). 3.09-Corrosion of magnesium and its alloys. Shreir’s Corrosion.

[13] Yang L., Huang Y., Feyerabend F., Willumeit R., Kainer K.U., Hort N. (2012). Influence of ageing treatment on microstructure, mechanical and bio-corrosion properties of Mg-Dy alloys. J. Mech. Behav. Biomed. Mater.

[14] Gray-Munro J.E., Seguin C., Strong M. (2009). Influence of surface modification on the *in vitro* corrosion rate of magnesium alloy AZ31. J. Biomed. Mater. Res. A.

[15] Hornberger H., Virtanen S., Boccaccini A.R. (2012). Biomedical coatings on magnesium alloys—A review. Acta Biomater.

[16] Yang J., Cui F., Lee I. (2011). Surface modifications of magnesium alloys for biomedical applications. Ann. Biomed. Eng.

[17] Shadanbaz S., Dias G.J. (2012). Calcium phosphate coatings on magnesium alloys for biomedical applications: A review. Acta Biomater.

[18] Badar M., Lünsdorf H., Evertz F., Rahim M.I., Glasmacher B., Hauser H., Mueller P.P. (2013). The formation of an organic coat and the release of corrosion microparticles from metallic magnesium implants. Acta Biomater.

[19] Bornapour M., Muja N., Shum-Tim D., Cerruti M., Pekguleryuz M. (2013). Biocompatibility and biodegradability of Mg-Sr alloys: The formation of Sr-substituted hydroxyapatite. Acta Biomater.

[20] Fan J., Qiu X., Niu X., Tian Z., Sun W., Liu X., Li Y., Li W., Meng J. (2013). Microstructure, mechanical properties, *in vitro* degradation and cytotoxicity evaluations of Mg-1.5Y-1.2Zn-0.44Zr alloys for biodegradable metallic implants. Mater. Sci. Eng. C.

[21] Gray-Munro J.E., Strong M. (2013). A study on the interfacial chemistry of magnesium hydroxide surfaces in aqueous phosphate solutions: Influence of Ca^2+^, Cl^−^ and protein. J. Colloid Interface Sci.

[22] Kannan M.B. (2012). Enhancing the performance of calcium phosphate coating on a magnesium alloy for bioimplant applications. Mater. Lett.

[23] Kim S.-M., Jo J.-H., Lee S.-M., Kang M.-H., Kim H.-E., Estrin Y., Lee J.-H., Lee J.-W., Koh Y.-H. (2014). Hydroxyapatite-coated magnesium implants with improved *in vitro* and *in vivo* biocorrosion, biocompatibility, and bone response. J. Biomed. Mater. Res. A.

[24] Iskandar M.E., Aslani A., Liu H. (2013). The effects of nanostructured hydroxyapatite coating on the biodegradation and cytocompatibility of magnesium implants. J. Biomed. Mater. Res. A.

[25] Johnson I., Akari K., Liu H. (2013). Nanostructured hydroxyapatite/poly(lactic-co-glycolic acid) composite coating for controlling magnesium degradation in simulated body fluid. Nanotechnology.

[26] Zomorodian A., Garcia M.P., Moura e Silva T., Fernandes J.C., Fernandes M.H., Montemor M.F. (2013). Corrosion resistance of a composite polymeric coating applied on biodegradable AZ31 magnesium alloy. Acta Biomater.

[27] Ostrowski N., Lee B., Enick N., Carlson B., Kunjukunju S., Roy A., Kumta P.N. (2013). Corrosion protection and improved cytocompatibility of biodegradable polymeric layer-by-layer coatings on AZ31 magnesium alloys. Acta Biomater.

[28] Kunjukunju S., Roy A., Ramanathan M., Lee B., Candiello J.E., Kumta P.N. (2013). A layer-by-layer approach to natural polymer-derived bioactive coatings on magnesium alloys. Acta Biomater.

[29] Killian M.S., Wagener V., Schmuki P., Virtanen S. (2010). Functionalization of metallic magnesium with protein layers via linker molecules. Langmuir.

[30] Wagener V., Killian M.S., Turhan C.M., Virtanen S. (2013). Albumin coating on magnesium via linker molecules—Comparing different coating mechanisms. Colloids Surf. B.

[31] Liu X., Yue Z., Romeo T., Weber J., Scheuermann T., Moulton S., Wallace G. (2013). Biofunctionalized anti-corrosive silane coatings for magnesium alloys. Acta Biomater.

[32] Ye S.-H., Jang Y.-S., Yun Y.-H., Shankarraman V., Woolley J.R., Hong Y., Gamble L.J., Ishihara K., Wagner W.R. (2013). Surface modification of a biodegradable magnesium alloy with phosphorylcholine (PC) and sulfobetaine (SB) functional macromolecules for reduced thrombogenicity and acute corrosion resistance. Langmuir.

[33] Drynda A., Hassel T., Hoehn R., Perz A., Bach F.-W., Peuster M. (2010). Development and biocompatibility of a novel corrodible fluoride-coated magnesium-calcium alloy with improved degradation kinetics and adequate mechanical properties for cardiovascular applications. J. Biomed. Mater. Res. A.

[34] Lozano R.M., Pérez-Maceda B.T., Carboneras M., Onofre-Bustamante E., García-Alonso M.C., Escudero M.L. (2013). Response of MC3T3-E1 osteoblasts, L929 fibroblasts, and J774 macrophages to fluoride surface-modified AZ31 magnesium alloy. J. Biomed. Mater. Res. A.

[35] Lin X., Tan L., Zhang Q., Yang K., Hu Z., Qiu J., Cai Y. (2013). The *in vitro* degradation process and biocompatibility of a ZK60 magnesium alloy with a forsterite-containing micro-arc oxidation coating. Acta Biomater.

[36] Wang Y.M., Guo J.W., Shao Z.K., Zhuang J.P., Jin M.S., Wu C.J., Wei D.Q., Zhou Y. (2013). A metasilicate-based ceramic coating formed on magnesium alloy by microarc oxidation and its corrosion in simulated body fluid. Surf. Coat. Technol.

[37] Levy G., Aghion E. (2013). Effect of diffusion coating of Nd on the corrosion resistance of biodegradable Mg implants in simulated physiological electrolyte. Acta Biomater.

[38] Weng L., Webster T.J. (2012). Nanostructured magnesium increases bone cell density. Nanotechnology.

[39] Weng L., Webster T.J. (2013). Nanostructured magnesium has fewer detrimental effects on osteoblast function. Int. J. Nanomed.

[40] Willumeit R., Fischer J., Feyerabend F., Hort N., Bismayer U., Heidrich S., Mihailova B. (2011). Chemical surface alteration of biodegradable magnesium exposed to corrosion media. Acta Biomater.

[41] Witte F., Kaese V., Haferkamp H., Switzer E., Meyer-Lindenberg A., Wirth C.J., Windhagen H. (2005). *In vivo* corrosion of four magnesium alloys and the associated bone response. Biomaterials.

[42] Bowen P.K., Drelich J., Goldman J. (2014). Magnesium in the murine artery: Probing the products of corrosion. Acta Biomater.

[43] Lorenz C., Brunner J.G., Kollmannsberger P., Jaafar L., Fabry B., Virtanen S. (2009). Effect of surface pre-treatments on biocompatibility of magnesium. Acta Biomater.

[44] Keim S., Brunner J.G., Fabry B., Virtanen S. (2011). Control of magnesium corrosion and biocompatibility with biomimetic coatings. J. Biomed. Mater. Res. B.

[45] Feyerabend F., Drücker H., Laipple D., Vogt C., Stekker M., Hort N., Willumeit R. (2012). Ion release from magnesium materials in physiological solutions under different oxygen tensions. J. Mater. Sci.: Mater. Med.

[46] Jerbi H., Hidouri M., Mongi B.A. (2012). Na7Mg13Nd(PO4)12. Acta Crystallogr. Sect. E.

[47] International A. (2012). Standard guide for laboratory immersion corrosion testing of metals. ASTM Standard NACE/ASTMG31-12a.

[48] Abele J.E. (1963). The physical background to freezing point osmometry and its medical-biological applications. Am. J. Med. Electron.

[49] Angermüller S., Fahimi H.D. (1982). Imidazole-buffered osmium tetroxide: An excellent stain for visualization of lipids in transmission electron microscopy. Histochem. J.

